# Cortical Network Synchrony Under Applied Electrical Field *in vitro*

**DOI:** 10.3389/fnins.2018.00630

**Published:** 2018-09-21

**Authors:** Min D. Tang-Schomer, Taylor Jackvony, Sabato Santaniello

**Affiliations:** ^1^Department of Pediatrics, UConn Health, Connecticut Children’s Medical Center, Farmington, CT, United States; ^2^The Jackson Laboratory for Genomic Medicine, Farmington, CT, United States; ^3^CT Institute for the Brain and Cognitive Sciences, University of Connecticut, Storrs, CT, United States; ^4^School of Medicine, UConn Health, University of Connecticut, Farmington, CT, United States; ^5^Biomedical Engineering Department, University of Connecticut, Storrs, CT, United States

**Keywords:** neural synchronization, neural interface, *in vitro* culture, neurostimulation, silk biomaterials, network analysis

## Abstract

Synchronous network activity plays a crucial role in complex brain functions. Stimulating the nervous system with applied electric field (EF) is a common tool for probing network responses. We used a gold wire-embedded silk protein film-based interface culture to investigate the effects of applied EFs on random cortical networks of *in vitro* cultures. Two-week-old cultures were exposed to EF of 27 mV/mm for <1 h and monitored by time-lapse calcium imaging. Network activity was represented by calcium signal time series mapped to source neurons and analyzed by using a community detection algorithm. Cortical cultures exhibited large scale, synchronized oscillations under alternating EF of changing frequencies. Field polarity and frequency change were both found to be necessary for network synchrony, as monophasic pulses of similar frequency changes or EF of a constant frequency failed to induce correlated activities of neurons. Group-specific oscillatory patterns were entrained by network-level synchronous oscillations when the alternating EF frequency was increased from 0.2 Hz to 200 kHz. Binary responses of either activity increase or decrease contributed to the opposite phase patterns of different sub-populations. Conversely, when the EF frequency decreased over the same range span, more complex behavior emerged showing group-specific amplitude and phase patterns. These findings formed the basis of a hypothesized network control mechanism for temporal coordination of distributed neuronal activity, involving *coordinated stimulation* by alternating polarity, and *time delay* by change of frequency. These novel EF effects on random neural networks have important implications for brain functional studies and neuromodulation applications.

## Introduction

Synchronized neural activities underlie many cognitive and behavioral responses during normal brain functioning (Buzsaki and Draguhn, 2004) and neurological disorders such as epilepsy (Burns et al., 2014; Yaffe et al., 2015) and schizophrenia (Uhlhaas and Singer, 2010). Neurons organize into functional networks that generate synchronized activities either spontaneously (Kirkby et al., 2013; Luhmann et al., 2016) or upon exogenous stimulus (Zhang and Poo, 2001; Tagawa et al., 2008). This process involves intrinsic molecular programs at the cellular level (Mathie et al., 2003; Holtmaat and Svoboda, 2009; Rebola et al., 2010; Bagley and Westbrook, 2012) and large scale (ensembles) information processing at the network level (Marom and Shahaf, 2002). Stimulating the central nervous system (CNS) with applied electric or magnetic field has become a common tool for probing neural networks in functional studies of the brain (Wagner et al., 2007; Bestmann et al., 2015). The applied electromagnetic fields affect CNS by generating a distributed electric field (EF) around the brain tissue underneath (McIntyre and Grill, 2002; Frohlich, 2014). Despite the wide-ranging neuro-modulatory effects of exogenous EF on the nervous system, the underlying mechanism for induced network changes remains elusive.

Major challenges for functional studies lie in the complexity of neural networks and the highly variable dynamics of neuronal responses. Neuronal response depends on the stimulus as well as the cell’s intrinsic properties. Studies (Lafon et al., 2017; Hutt et al., 2018) have recently shown that high-frequency periodic stimulation can lead to waveform-dependent changes in the oscillatory dynamics of neurons while randomly fluctuating stimulation linearizes the neuron response function. Other studies, instead, have shown that the neuron’s response change as a function of the intensity, duration, and polarity of the stimulus (McIntyre and Grill, 2002; Wagenaar et al., 2004) and these changes are noticed at multiple spatial scales, from small neuronal clusters to large populations (Yoo et al., 2004; Yu et al., 2017, 2018). Neuronal sensitivity depends on the cell’s channel protein and receptor composition, synapse maturate state, and cell morphology (O’Brien et al., 1998; Mathie et al., 2003; Holtmaat and Svoboda, 2009; Rebola et al., 2010; Bagley and Westbrook, 2012; Yi et al., 2017). Models with defined network architecture and cell compositions, such as *ex vivo* brain slices or specific CNS pathways, are used to determine conditions capable of evoking functionally relevant responses. For example, continuous stimulation at 5 Hz corresponds to the resting state at hippocampal synapses (Bito et al., 1996) and context-dependent stimuli mimics neuronal activities during learning (Larson et al., 1986; O’Keefe and Recce, 1993; Staubli et al., 1999). However, it is unclear how those region- or pathway-specific findings can be applied elsewhere in the CNS.

*In vitro* cultures of dissociated neurons combined with multi-electrode arrays (MEAs) provide an alternative model for studying neural networks. Such cultures retain many *in vivo* features, including connectivity and cell type distribution, as well as synaptic and cellular level plasticity, e.g., see (Marom and Shahaf, 2002) for a review. *In vitro* cortical cultures allow much more detailed observation and manipulation than intact brains. Cultures exhibit spontaneous periodic calcium transients or bursting activities (Robinson et al., 1993; Maeda et al., 1995; Jimbo et al., 2000; Opitz et al., 2002), with increased propensity for synchronized bursting as the culture matures (Kamioka et al., 1996; Tateno et al., 2002; Sun et al., 2010). Interrogated by site-specific stimuli with varying temporal and spatial features, *in vitro* cortical networks exhibit *in vivo*-relevant adaptive behavior (Eytan et al., 2003). Studies have shown pathway-specific (rather than neuron-specific) changes in neuronal responsiveness, including potentiation or depression (Jimbo et al., 1999), and stimuli context-dependent plasticity (Bakkum et al., 2008a). Network-level signal propagation involves intrinsic firing of random neurons, recruitment of other neurons, and repetitive excitation leading to synchronous burst firing (Jimbo et al., 2000; Chao et al., 2007; Bakkum et al., 2008a; Kryukov et al., 2008; Manyakov and Van Hulle, 2008; Sun et al., 2010; Yu et al., 2011a,b, 2012, 2013). These studies have led to attempts to develop a self-learning network with *in vitro* cortical cultures, for example, with a closed-loop training algorithm to guide the network toward a pre-determined activity state (Wagenaar et al., 2005; Bakkum et al., 2008b), and with patterned stimuli to increase burst firing probability of a selective loci (Shahaf and Marom, 2001). However, there are considerable viabilities regarding the design of MEA systems, the network topology and variable endogenous activities of heterogeneous neuronal populations (Morin et al., 2005). In this study, we sought to identify stimulation conditions that can induce synchronized activities of a random network of *in vitro* cortical cultures. To avoid some of the model-specific features with point stimulation, we applied a uniform field with substrate-embedded electrode pair spanning the culture.

An *in vitro* cortical culture has a small world topology (Watts and Strogatz, 1998) characterized by dense local clustering of neighboring nodes (neurons) and a short path length (axon connections) between any pair of nodes. Since small-world network architecture is widespread in biological neural networks, properties of the living neuronal network will have broad implications to other nervous systems (Bassett and Bullmore, 2006). The effect of stimulations (including electric and magnetic fields) on synchrony dynamics of nervous system has been extensively studied in mathematics and computational neuroscience (Yu et al., 2013). These theoretical studies have produced mechanistic insights regarding network dynamics, for example, chaos phase locking (Yu et al., 2011b), time-delayed feedback for synchrony suppression (Yu et al., 2013), simulated tACS effects (Ali et al., 2013; Frohlich, 2015). However, the insights gained from such modeling strategies can only be fully leveraged when used in conjunction with experimental approaches. Our goal is to develop a cell culture-based model with quantifiable and controllable population-wide synchronous activities to be used for testing network theories.

Population-wide analysis of neuronal activities requires the detection of families of neurons having a similar activity pattern, so that the original neuronal network can be decomposed into distinct clusters. With electrical recordings, algorithms are needed for detecting bursts and defining their attributes (e.g., duration) as unitary events, and for correlation analysis of the time series of bursts (Parodi et al., 1998; Chao et al., 2007; Sun et al., 2010). Alternatively, calcium live imaging can be used to monitor large populations of neurons within a field of view simultaneously. Synchronized calcium transients are direct result of propagation of bursts of action potentials that are generated periodically by *in vitro* cortical cultures (Robinson et al., 1993). When mapped onto the source neurons, calcium time series allow for direct comparison of the temporal and spatial patterns of neuronal activities. In this study, we developed computational analysis of calcium signals based on graph theory and network community detection to identify functionally correlated neuronal clusters. We tested a local greedy-optimization algorithm (Blondel et al., 2008) to automatically determine the best partition of the neuronal population (i.e., number of communities and composition of each detected community) with minimal computational cost. Communities returned by the algorithm are entirely based on calcium signals and therefore capture a common behavior across neurons.

The experimental setup of this study built upon a previously developed neural-electric interface, consisting of dissociated cortical neurons growing on a silk fibroin film with embedded gold wires (Tang-Schomer et al., 2014a,b). Silk fibroin-based biomaterial has found extensive applications in neural engineering, as biosensors (Domachuk et al., 2009; Kim et al., 2010), neural probes (Kim et al., 2009; Tien et al., 2013), and for tissue engineering of the nervous system (Tang-Schomer et al., 2014c; Chwalek et al., 2015a,b). The silk film-based neural-electric interface has shown evoked calcium influx of *in vitro* cultured rat cortical neurons by applied EF (Tang-Schomer et al., 2014a,b). This study examined cortical network activities under different stimulus patterns, varying in frequencies and directions, and evaluated temporal and spatial associations of neuronal populations. The findings revealed novel EF effects on random neural networks and provided guiding principles for control of network synchrony *in vitro*.

## Materials and Methods

### Silk Film Supported Neural-Electric Interface

Silk films were processed as previously reported in (Tang-Schomer et al., 2014b). Briefly, silk fibroin (1–2%) solution extracted from *Bombyx mori* silkworm cocoons (Tajima Shoji Co., Yokohama, Japan) was prepared. A pair of gold wires (100 μm diameter, SPM Inc., Armonk, NY, United States) were positioned at 6 mm apart onto a PDMS mold (16 mm diameter) and immersed in silk solution. After drying in air, the silk film (∼5 μm thickness) was peeled off the mold with the gold wires embedded in the film. Films were rendered water-insoluble by β-sheet formation via water annealing in a water-filled desiccator for >5 h. To prepare for cell culture, the film was UV sterilized, coated with 20 μg/ml poly-L-lysine (Sigma-Aldrich, St. Louis, MO, United States) overnight, washed and dried prior to introducing cells.

### Primary Cortical Neuronal Culture

The rat brain tissue dissociation protocol was approved by Tufts University Institutional Animal Care and Use Committee and complies with the NIH Guide for the Care and Use of Laboratory Animals (IACUC # B2011-45). Cortices from embryonic day 18 (E18) Sprague Dawley rats (Charles River, Wilmington, MA, United States) were isolated, dissociated with trypsin (0.3%, Sigma) and DNase (0.2%, Roche Applied Science, Indianapolis, IN, United States) followed with trypsin inhibition with soybean proteins (1 mg/mL, Sigma), centrifuged, and plated at 200,000–625,000 cells/cm^2^ in neuro-basal media (Invitrogen, Carlsbad, CA, United States) supplemented with B-27 neural supplement, penicillin/streptomycin (100 U/mL and 100 μg/mL), and GlutaMax (2 mM, Invitrogen). Cultures were maintained in 37°C, 100% humidity and 5% CO_2_ in an incubator (Forma Scientific, Marietta, OH, United States) for up to 16 days *in vitro* (DIV 1–16). Cultures of DIV 14–16 were used for stimulation.

### Electrical Stimulation

The interface cultures were set up with extensions of the silk protein film-embedded gold wires connected to an electrical stimulator, as previously described in (Tang-Schomer et al., 2014b). The field potential was set at 160 mV between the electrodes and validated with an oscilloscope. A functional generator (Tenma Universal Test Center 72-5085, MCM Electronics, Centerville OH, United States) delivered biphasic, rectangular waves with frequencies ranging from 0.2 Hz to 200 kHz. Monophasic pulses (0.1 ms) were delivered by a Grass S44 stimulator and SIU5 stimulation isolation unit at frequencies ranging from 0.2 Hz to 2 kHz. A total of 12 cultures from six independent batches of cells (i.e., rats) were used. Voltage applied across each silk film was verified prior to stimulation with an oscilloscope. No cellular damage was observed during all our experiments, based on morphological characterization.

### Calcium Imaging and Image Analysis

Calcium dye Fluo-4 AM (Invitrogen) was used to visualize changes in intracellular calcium concentration. Calcium imaging with fluorescent calcium indicators is a reliable method to monitor action potential activities (Burnett et al., 2003), as intracellular calcium concentration rises transiently during electrical activity to levels that are 10–100 times higher (Berridge et al., 2000). Calcium imaging of bulk-loaded fluorescent indicators can be used to record the spiking activity of hundreds of neurons (Kwan, 2008).

Experiments were performed in controlled saline solution (CSS: 120 mM NaCl, 5.4 mM KCl, 0.8 mM MgCl_2_, 1.8 mM CaCl_2_, 15 mM glucose, and 25 mM HEPES, pH 7.4). Cultures were loaded with 1 μg/ml dye solution (in PBS containing 0.2% DMSO) at 37°C for 30 min, washed with PBS, and incubated in fresh media for another 30 min. The cultures were mounted onto a confocal microscope (Leica TCS SP2, Leica Microsystems, Wetzlar, Germany) within an environmental chamber with the temperature maintained at 37°C.

During stimulation, time-lapse fluorescence images were acquired with the same optical settings (at Ex/Em of 488/525 nm). For field stimulation, we imaged a 30-section *z*-stack every minute for 45–60 min. Time-series fluorescence images of one focal plane at the middle-point of a *z*-stack were used for image analysis. For pulse stimulation, images at a fixed focal plane were acquired every 10 s (i.e., Δ*t* = 10 s) for 20–30 min.

NIH Image J software suite was used to quantify the fluorescence intensity. Circular selection was made for each cell body, and the mean fluorescence intensity was measured. A neuron’s fluorescence intensity at a specific time point *t* (*F*_t_) divided by the intensity at time 0 (*F*_0_, no stimulation) of the same neuron gave the calcium signal change and reported as *F*_t_/*F*_0_.

### Network Analysis and Unsupervised Community Detection

For the analysis of the functional connectivity between neurons, a.k.a. network analysis (Newman, 2010), sample distribution of the fluorescence intensities at time 0 (*F*_0_) was estimated and the values of mean (μ_0_) and standard deviation (σ_0_) were computed. Each cortical culture was assumed to be representative of *all* cortical cultures, as a common practice with primary cortical culture-based studies. To further compare different cell cultures, we normalized each fluorescence measurement against the global average. Specifically, each fluorescence intensity time series *F*_t_ was normalized by subtracting μ_0_ and dividing by σ_0_. This normalization procedure aimed at preserving the range of fluorescence intensities observed across each cortical culture while removing time-series-specific biases. The normalized fluorescence intensity time series were then used to run the network partition algorithm described in section 3 and to identify functional clusters.

A local greedy-optimization algorithm was used to automatically determine the best partition of the neuronal population (i.e., the best number of communities and composition of each community) with minimal computational cost. We defined the communities returned by the algorithm as functional clusters as the neurons within the community had fluorescence time series with high degree of temporal correlation. Specifically, we envisioned each neuron in the culture as a node in a fully connected network, i.e., we assumed an edge between nodes *i* and *j*, for all *i*, *j* = 1, 2, 3, …, *N*, where *N* is the number of labeled neurons in the culture. For each pair (*i*, *j*), a weight *w_i,j_* was assigned to the edge between *i* and *j*, with *w_i,j_* being the Pearson correlation coefficient between the normalized fluorescence intensity time series estimated for neuron *i* and neuron *j*, respectively. The functional network is univocally defined by the weighted adjacency matrix (Newman, 2010).

(1) $$𝒜=\left[\begin{array}{llll} 0 & w_{1,2} & \cdots & w_{1,N}\\ w_{2,1} & 0 & \cdots & w_{2,N}\\ \vdots & \vdots & \ddots & \vdots \\ w_{N,1} & w_{N,2} & \cdots & 0 \end{array}\right]$$

which is a *N × N* symmetric matrix and has zeros on the main diagonal because no node forms edges with itself. We applied a static community detection algorithm (Newman, 2010) on matrix 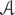 to identify meaningful group structures in the neuronal network. A community is a set of nodes (i.e., cultured neurons) that are connected among one another more densely than they are to nodes in other communities, and nodes within a community may share similar structural or functional properties (Newman, 2010).

We used the Louvain algorithm (LA) (Blondel et al., 2008) to partition matrix 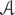 in communities. Briefly, LA identifies communities in a network by optimizing a quality function known as “modularity index”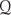 (Newman, 2010), which measures the density of edges inside the communities compared to edges between communities. Communities are estimated by comparison between the assigned network and a null model (Newman-Girvan null model) (Newman, 2010) and high modularity index values indicate large separation between communities. Because LA is a locally greedy optimization algorithm, we ran the community detection procedure for a total of 100 optimizations and used a consensus partition method (Lancichinetti and Fortunato, 2012) to obtain a consistent community partitioning in each network. After the functional clusters were determined, individual neurons were color-coded accordingly onto the original fluorescence image, to compare with their physical partitioning.

## Results

**FIGURE 1:**
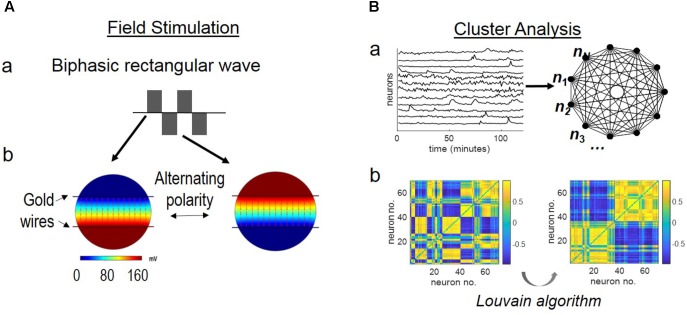
Schematics of stimulation setup and network analysis. **(A)** Field stimulation. Stimulus was delivered by substrate-embedded gold wires spanning the cortical culture. **(a)** A biphasic rectangular wave. **(b)** COMSOL simulation of the electric field (160 mV peak-to-peak amplitude, 6 mm-apart electrode distance), as previously described (Tang-Schomer et al., 2014b). Field distribution at the positive and negative phases of the wave is shown. **(B)** Cluster analysis. **(a)** Labeled neurons *n*_1_, *n*_2_, …, *n_N_* are envisioned as the nodes of an all-to-all graph (*right*) and the connection between any two neurons *n_i_*, *n_j_* is weighted by the Pearson correlation coefficient *w_i,j_* between the correspondent fluorescence intensity time series (*left*). **(b)** Correlation-based weighted adjacency matrix 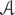 before (*left*) and after (*right*) sorting the neurons according to the community partition given by the Louvain algorithm (LA). Color-map reports the range of correlation coefficients.

An electrical field was imposed to the cortical neurons by a pair of substrate-embedded gold wires spanning the *in vitro* culture (**Figure 1A**). **Figure 1A-a** shows a biphasic, rectangular wave. **Figure 1A-b** shows the simulated EF distribution by the COMSOL software, as described previously in (Tang-Schomer et al., 2014b). The biphasic wave introduced EF of alternating polarity during the positive and negative phases of the wave function, at the rate of the wave frequency.

In conventional stimulation experiments, the parameters of stimulus (amplitude, frequency, duration) are determined by paring with intracellular recording of evoked responses of targeted neurons (Jayakar et al., 1992; Bagley and Westbrook, 2012). This study used voltage (160 mV across 6 mm) that showed frequency-dependent calcium responses of cortical neurons in a similar system (Tang-Schomer et al., 2014a,b). Our setup generated a theoretical EF strength of 27 mV/mm, above the threshold extracellular voltage gradient of 5–10 mV/mm for evoked neuronal response (Jefferys, 1995) (**Figure 1B**).

### Network Synchronization Under Alternating EF With Increasing Frequencies

When we monitored cortical cultures without stimulation for 10 min a time, no oscillatory calcium responses were found, and calcium signals fluctuated within 20% of the baseline level. Cortical cultures under alternating EF of a constant frequency (i.e., 2 and 10 Hz) also failed to produce synchronized activities. Only square waveform was tested in the study.

**FIGURE 2:**
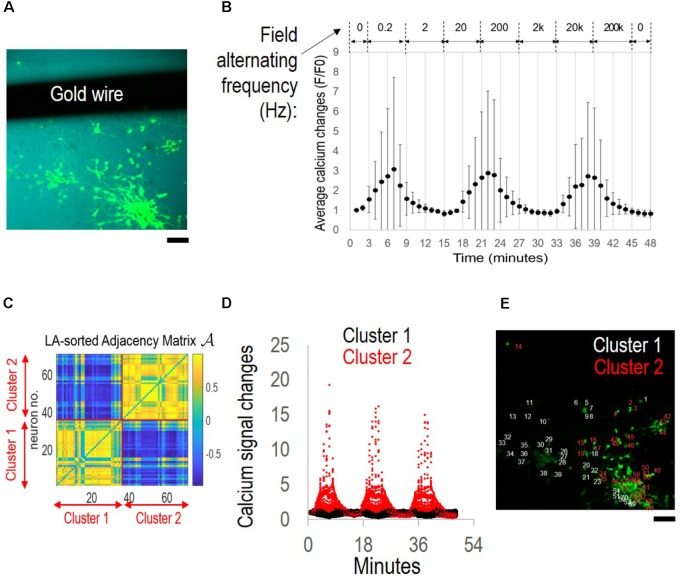
Network synchronization under alternating field with increasing frequencies. The cortical culture was exposed to an alternating field with frequencies increasing from 0.2 Hz to 200 kHz for 6 min per condition. **(A)** Fluorescence image of fluo-4 stained neurons overlay with bright field image. The dark area is the film-embedded gold wire. Scale bar, 100 μm. **(B)** Average calcium signal time series from 70 neurons. Error bar depicts the standard derivation. **(C)** Correlation-based weighted adjacency matrix 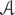 after sorting the neurons according to the community partition given by the LA. Two clusters were obtained. Red lines mark the separation between Cluster 1 and Cluster 2. Color bar reports the range of Pearson’s correlation coefficient values. **(D)** Functional clusters color-coded onto the calcium signal time series. Cluster 1 (non-responders) in *black*, and Cluster 2 (super-responders) in *red*. **(E)** Functional clusters mapped onto the original fluorescence image of the culture. Neurons in Cluster 1 (non-responders) in *white*, and those in Cluster 2 (super-responders) in *red*. Scale bar, 100 μm.

To our surprise, when biphasic, rectangular waves with field polarity alternating from 0.2 Hz to 200 Hz were applied, large-scale, synchronized oscillations of cortical neurons were observed (**Figure 2**). **Figure 2A** shows the example of neurons stained with fluo-4, a calcium indicator, adjacent to a silk film-embedded gold wire electrode (field of view, 750 by 750 μm).

**Figure 2B** shows the stimulation protocol of alternating EF with increasing frequencies and the average calcium signal time series of the cortical culture. The experiment was conducted in a temperature controlled (37°C) environmental chamber and lasted for less than 1 h. Stimulus was introduced at the third minute of live imaging and increased from 0.2 Hz by 10-fold a time to 200 kHz for 6 min per condition. The average calcium signal showed synchronous oscillations of approximately 15 min wave length (70 neurons measured).

The community detection algorithm was used to sort the neurons based on the statistical significance of the differences of their calcium signals, and two clusters were identified (**Figure 2C**). Neurons in the same cluster were highly correlated (i.e., Pearson’s correlation coefficient >0.5) (*yellow*). Neurons belonging to different clusters were either poorly correlated (i.e., Pearson’s correlation coefficient close to 0) or negatively correlated (i.e., Pearson’s correlation coefficient close to −1) (*blue*).

Calcium signal time-series were then color-coded (**Figure 2D**) according to whether they referred to neurons in Cluster 1 (*black*) or Cluster 2 (*red*). Cluster 1 contained “*non-responders*” with calcium signals fluctuating close to the baseline level. Cluster 2 contained “*super-responders*” with calcium signal increases >5-folds of the baseline level.

When mapped onto the original image, the functional clusters found remarkable match with the neurons’ physical partitioning (**Figure 2E**). Neurons belonging to the same functional cluster resided in close proximity to each other and separate from neurons belonging to the other cluster (Cluster 1, non-responders, *white*; Cluster 2, super-responders, *red*).

### Entrainment of Sub-populations’ Oscillations by Network Synchronization

**FIGURE 3:**
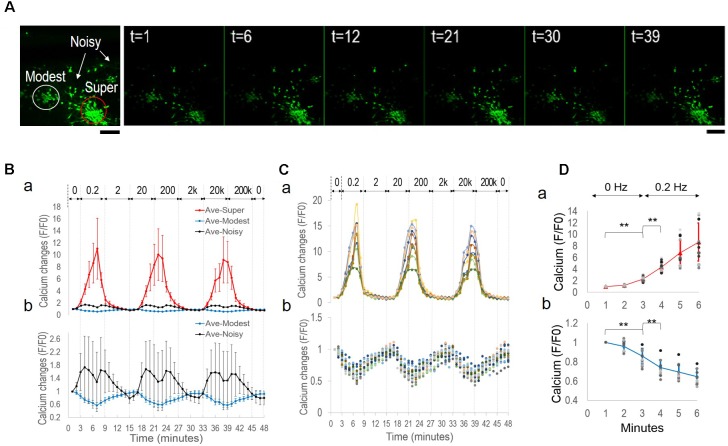
Oscillatory patterns of neuronal subpopulations under alternating EF of increasing frequencies. **(A, a)** Neurons sub-divided into groups of super-responders (*Super*), modest responders (*Modest*), and noisy responders (*Noisy*). **(b)** Representative fluorescence images of fluo-4 stained neurons at specified time points (in minutes). Note the differences of different groups’ intensity changes. Scale bar, 100 μm. **(B)** Average calcium signal time series from each sub-population. **(a)** super-responders (*red*), modest-responders (*blue*), and noisy-responders (*black*). **(b)** Magnified plots of modest-responders (*blue*) and noisy-responders (*black*). Error bar depicts the standard derivation. **(C)** Individual calcium signal time series of super-responders **(a)** and modest-responders **(b)**. **(D)** Calcium signal time series of the first 6 min of stimulation experiment, 0–3 min no stimulation and transition to 0.2 Hz from the third minute. **(a)** Super-responders. **(b)** Modest-responders. Error bar depicts the standard derivation. ANOVA test, ^∗∗^*P*-value *P <* 0.01.

By manual examination of calcium signal traces, we further divided the apparent non-responders into two groups, i.e., “*modest-responders*” with <5-fold signal changes but displaying synchronized activities and the rest as “*noisy-responders*.” When mapped onto the original image, these sub-populations were found to belong to distinctive physical groups (**Figure 3A-a**): the super-responders and modest-responders as two separate neuronal aggregates (in *red* and *white* circles, respectively), and the noisy-responders consisting cells dispersed in the surrounding areas (*arrows*). **Figure 3A-b** displays representative images at specific time-points (in minute), demonstrating different fluorescence changes of neuronal sub-populations.

**Figure 3B** shows the average calcium time series from super-responders (*red*, *n* = 14, 20%), modest-responders (*blue*, *n* = 17, 24%), and noisy-responders (*black*, *n* = 39, 56%). The super-responders had peak signal levels of approximately 10-fold increases, compared to <2-fold changes of the other groups (**Figure 3B-a**). Further close examination of the low-amplitude signal changes of the modest-responders and noisy-responders revealed that they, too, exhibited synchronized oscillations (**Figure 3B-b**). Notably, all three sub-populations’ oscillatory patterns were entrained by the network-level synchronous oscillation. The sub-population showed group-specific amplitude and phase patterns. For example, the modest-responders (*blue*) had opposite phase responses than the super-responders (*red*), i.e., peaks in one plot correspond to troughs in the other plot and vice versa. The noisy-responders’ signal trace (*black*) had its major peaks in phase with other sub-populations but contained two smaller peaks.

### Symmetrical Phase Changes and Dependence on Group-Specific Spontaneous Activities

To understand the phase differences between the super-responders and modest-responders, we examined individual calcium time series (**Figure 3C-a**: super-responder; **Figure 3C-b**: modest-responder). The traces showed remarkable synchrony within each sub-population. The synchronous oscillations of the two groups exhibited opposite phase changes.

We then focused our analysis on the initial period of the experiment, i.e., when the culture was switched from being un-stimulated for the first 3 min to 0.2 Hz alternating EF for another 6 min. **Figure 3D** shows the average calcium signal time series of the first 6 min (a, super-responder; b, modest-responder). The two sub-populations had opposite activity trends prior to stimulation, with spontaneous calcium signal increases and decreases, respectively. There were significant differences of their signal levels at the third minute compared to the first minute. Upon stimulation, the different calcium responses continued their opposite trajectory that were further enhanced by the 0.2 Hz EF. Significant differences were observed in the signal level at 1-min post-stimulation (i.e., the fourth minute) compared to right before the stimulation (i.e., the third minute).

The above findings showed that a random network of cortical culture contained sub-populations of distinctive physical partitioning and endogenous activity levels. Alternating EF of increasing frequencies induced synchronization within each sub-population as well as across the entire network, while retaining group-specific oscillatory patterns. The binary response of activity-increase or decrease contributed to the opposite phase patterns of different sub-populations.

### Symmetrical Sub-population’s Oscillatory Patterns Under Alternating EF With Decreasing Frequencies

**FIGURE 4:**
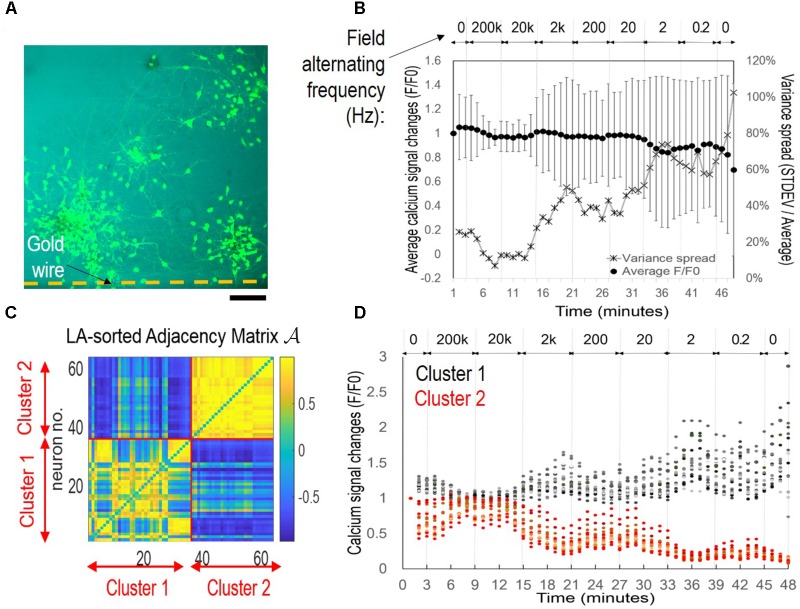
Symmetrical sub-population’s oscillatory patterns under alternating EF with decreasing frequencies. The cortical culture was exposed to an alternating field with frequencies decreasing from 200 kHz to 0.2 Hz for 6 min per condition. **(A)** Fluorescence image of fluo-4 stained neurons overlay with bright field image. The substrate-embedded gold wire is right blow the imaged area outside the field of view. Scale bar, 100 μm. **(B)** Average calcium signal time series from 63 neurons (*dot*), and variance spread defined as standard derivation divided by the mean (*cross*). Note the large error bar (standard derivation) in lower frequencies. **(C)** Correlation-based weighted adjacency matrix 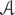 after sorting the neurons according to the community partition given by the LA. Two clusters were obtained. Red lines mark the separation between Cluster 1 and Cluster 2. Color bar reports the range of Pearson’s correlation coefficient values. **(D)** Functional clusters color-coded onto the calcium signal time series. Cluster 1 is reported in *black* and Cluster 2 in *red*. Note the symmetry of their signal patterns.

Considering that applied EF of a constant frequency failed to induce network synchronization, we suspected that the context of EF frequency change was critical. We therefore conducted a different experiment, in which a different cortical culture was exposed to alternating EF of decreasing frequencies (**Figure 4**). We applied biphasic, rectangular waves (peak-to-peak 160 mV) with frequencies starting from 200 kHz at the third minute and decreased by 10-fold to 0.2 Hz for 6 min per condition. **Figure 4A** shows the fluo-4 stained neurons adjacent to a silk film-embedded gold wire; the wire was right blow the imaged area outside the field of view.

**Figure 4B** shows the average calcium time series (*black dots*) from 63 neurons measured. The mean activity level appeared to be mostly flat with a down-ward trend, with large variance of each data point. When we plotted the variance spread (**Figure 4B**, *crosses*), measured as the ratio of standard derivation to the mean, a dependence on EF frequency was found. The baseline variance of 26% decreased to 7% after 5 min of 200 kHz stimulation, maintained at approximately 12% during 20 kHz stimulation, and rose progressively as the frequency decreased, until reaching 102% at the end of the experiment (total time < 1 h). These results suggested that there were mixed responses of different sub-populations, and functional association of these groups depended on EF frequency change.

We used the community detection algorithm to automatically group the 63 neurons into two functional clusters (**Figure 4C**). Neurons within a cluster were highly correlated and poorly or negatively correlated to neurons in the other cluster, thus reflecting a marked functional separation between clusters. Color-coded calcium signal time series in **Figure 4D** revealed cluster-specific signal patterns that were previously obscured in the total average trace (**Figure 4B**). Cluster 1 (*black*) neurons had increased activity and Cluster 2 (*red*) neurons decreased activity. Notably, there was symmetry of plots between the two groups with peaks in one group corresponded to troughs of the other group.

### Suppression of Spontaneous Activity by High Frequency Alternating EF

**FIGURE 5:**
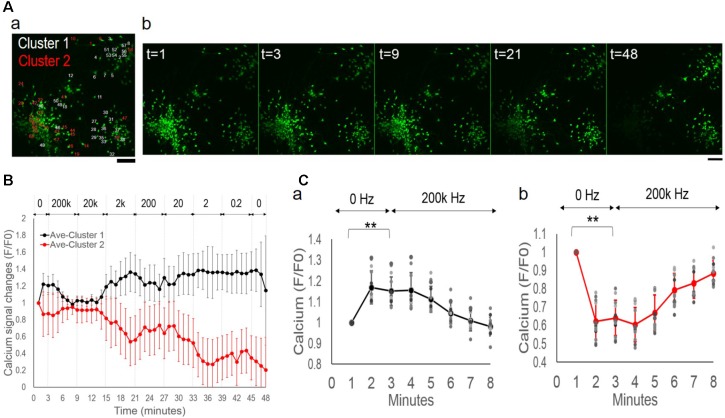
Inhibition of spontaneous activity by high frequency alternating EF. **(A, a)** Functional clusters mapped onto the original fluorescence image of the culture under alternating EF with decreasing frequencies. Neurons in Cluster 1 are in *white* and neurons in Cluster 2 are in *red*. **(b)** Representative fluorescence images of fluo-4 stained neurons at specified time points (in minutes). Note the differences of different groups’ intensity changes. Scale bar, 100 μm. **(B)** Average calcium signal time series from Cluster 1 (*black*) and Cluster 2 (*red*). Error bar depicts the standard derivation. **(C)** Calcium signal time series of the first 6 min of stimulation experiment, 0–3 min no stimulation and transition to 200 kHz from the third minute. **(a)** Cluster 1. **(b)** Cluster 2. Error bar depicts the standard derivation. ANOVA test, ^∗∗^*P*-value *P <* 0.01.

To better understand the differences between Cluster 1 and Cluster 2, we mapped individual neurons onto the original image (**Figure 5A-a**, Cluster 1 in *white*. Cluster 2 in *red*). The functional clusters matched neuronal physical groups, as neurons belonging to the same cluster were in proximity to one other and separate from the other cluster. **Figure 5A-b** displays representative images at specific time-points, demonstrating different fluorescence changes of neuronal sub-populations.

**Figure 5B** shows the average calcium time series of the two clusters (Cluster 1, *black*. Cluster 2, *red*) and demonstrates group-specific oscillatory patterns. Both clusters started with opposite spontaneous activity changes (increase versus decrease), had suppressed activities during 200 kHz and 20 kHz stimulation, and continued with opposite activity changes in terms of amplitude and phase patterns as the frequency decreased. The average time series in Cluster 1 and Cluster 2 had significantly different trends (two-way ANOVA test with cluster label and time as factors, *P*-value *P* < 10^−10^ for the cluster factor). Moreover, for each frequency depicted in **Figure 5B**, we tested whether the responses of Cluster 1 and Cluster 2 were significantly different (Wilcoxon Rank-Sum test, *P*-value *P* < 0.05) and we found that the responses during stimulation at 200 Hz, 20 Hz, 2 Hz, and 0.2 Hz were significantly different. Finally, we looked at the sample distribution of the correlation coefficient of the normalized fluorescence signals in Cluster 1 and Cluster 2 and we found that the distributions were significantly different (0.18 ± 0.60 versus 0.22 ± 0.88, Cluster 1 versus Cluster 2, mean ± S.D, Wilcoxon Rank-Sum test, *P*-value *P* < 10^−10^). The sample distribution of the correlation coefficient was uniformly distributed between −1 (anti-phase) and +1 (in-phase) for Cluster 1 while it was polarized around −1 and +1 (bimodal distribution) for Cluster 2, thus indicating a stronger level of intra-cluster correlation for Cluster 2.

We then examined the differences of the two sub-populations during the initial period of the experiment (**Figure 5C**), when the culture was switched from being unstimulated for the first 3 min to under 200 kHz alternating EF stimulation for another 6 min. Cluster 1 (**Figure 5C-a**) and Cluster 2 (**Figure 5C-b**) neurons had calcium signal increase of 15 ± 6% (*n* = 33, *P*-value *P <* 0.01) and decrease of 36 ± 10% (*n* = 30, *P*-value *P <* 0.01), respectively, at the third minute compared to the first minute. Upon stimulation of 200 kHz alternating EF, the opposite calcium signaling trends were attenuated, and both sub-populations headed toward the baseline level.

### Network Desynchronization Under Alternating EF With Decreasing Frequencies

**FIGURE 6:**
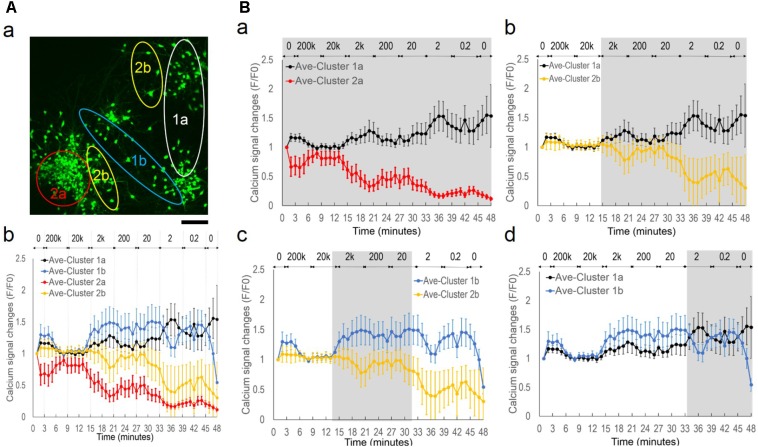
Network desynchronization under alternating EF with decreasing frequencies. **(A**, **a)** Functional clusters 1a, 1b, 2a, and 2b mapped onto the original fluorescence image of the culture under alternating EF with decreasing frequencies. Scale bar, 100 μm. **(b)** Average calcium signal time series of each sub-population. Legend: Cluster 1a, *black*; Cluster 1b, *blue*; Cluster 2a, *red*; Cluster 2b, *yellow*. **(B)** Pair-wise comparison of sub-population’s calcium signal time series. **(a)** Cluster 1a versus Cluster 2a. **(b)** Cluster 1a versus Cluster 2b. **(c)** Cluster 1b versus Cluster 2b. **(d)** Cluster 1a versus Cluster 1b. Gray background highlights the symmetrical areas of the plots.

By closer examination of each neuron’s activity, we further manually divided the clusters into four groups based on similarities of their fluorescence time series. This manual process re-grouped the neurons with subjectively determined similarities of the calcium signals, with no exclusion or other assumptions, i.e., clusters 1a (*n* = 20, 32%), 1b (*n* = 13, 21%), 2a (*n* = 16, 25%), 2b (*n* = 14, 22%) (**Figure 6**). **Figure 6A-a** shows the general distribution of the sub-populations. Cluster 1a and 2a contained two well-separated neuronal aggregates. Cells interspaced in surrounding areas were contained in Cluster 1b and Cluster 2b. **Figure 6A-b** shows each cluster’s average calcium signal time series. The variance at each data point remained consistent within each group in contrast to the highly variable total average response in **Figure 4B**, indicating similar intra-group but different inter-group signal patterns. All clusters showed suppressed activities under 200 kHz and 20 kHz stimulation. However, starting from 2 kHz, there was great divergence of activity trends with group-specific oscillatory patterns as the frequency decreased.

**Figure 6B** shows pair-wise comparison between the clusters of calcium signal time series. An opposite trend was observed between the sub-population-specific oscillatory patterns, as highlighted in gray (Wilcoxon Rank-Sum test, *P*-value *P* < 0.05). Cluster 1a showed phase symmetry (i.e., peak versus trough) and opposite activity changes (i.e., increase versus decrease) with Cluster 2a (**Figure 6B-a**) and Cluster 2b (**Figure 6B-b**) under all frequencies (2000–0.2 Hz). Cluster 1b showed symmetric phase and activity changes in specific frequency ranges, with Cluster 2b between 2000 and 20 Hz (**Figure 6B-c**) and Cluster 1a at ≤2 Hz (**Figure 6B-d**).

Taken together, these behaviors suggested a network de-synchronization process. The initial globally suppressed network diverged into two groups, Cluster 1 and Cluster 2 with opposite activity trends and phase patterns. As the alternating EF frequency decreased, neurons in Cluster 2 split into subgroups of 2a and 2b with oscillations of synchronized phase patterns but different amplitudes. Neurons in Cluster 1 split into subgroups of 1a and 1b that initially had synchronized phase patterns and different amplitudes, but under further decreased EF frequency, exhibited opposite phase patterns.

### Lack of Synchronized Activity Under EF Without Polarity Change or Continuous Frequency Change

**FIGURE 7:**
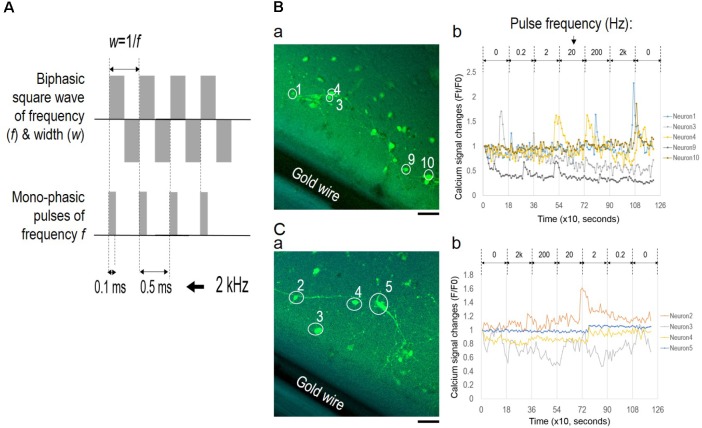
Lack of network synchronization under monophasic EF. **(A)** Wave function comparison of biphasic square wave and monophasic pulses of the same frequency. The monophasic pulse (0.1 ms) trains capture the initial field changes of each positive phase of the corresponding biphasic wave. **(B)** Fluorescence images of neurons **(a)** and corresponding calcium time series **(b)** under pulse trains of increasing frequencies. **(C)** Fluorescence images of neurons **(a)** and corresponding calcium time series **(b)** under pulse trains of decreasing frequencies. Only neurons with significant spiking activities are shown in the calcium time series and marked onto the corresponding images. Scale bar, 100 μm.

To examine the role of EF polarity in network synchrony, we designed a different set of stimulation experiments with monophasic EF of similar frequency changes as the alternating EF (**Figure 7**); different batches of cortical cultures were used. **Figure 7A** shows wave function comparison of biphasic EF and monophasic pulse trains of a fixed 0.1 ms pulse duration. The pulse train captured the initial moment of field potential change upon each stimulus at the same frequency as the corresponding biphasic waves. However, the pulse trains lacked field polarity change of the biphasic waves.

The pulse train was delivered at frequencies ranging from 0.2 Hz to 2 kHz for 3 min for each condition, and calcium fluorescence images were collected every 10 s. **Figures 7B,C** show fluorescence images of neurons (a) and corresponding calcium time series (b) under conditions of increasing frequencies and decreasing frequencies, respectively. In both scenarios, most of the neurons showed activity fluctuation within 20% of the baseline levels, and only selective neurons reported spiking activities as shown in **Figures 7B-b,C-b** (non-spiking activities were omitted). Statistical analysis of fluorescence intensity time series from individual neurons determined that neuronal activities in both scenarios were largely uncorrelated. Pearson’s correlation coefficients between spiking neurons were close to 0, indicating that these neurons activated independently from one another.

In another set of experiments, we examined the role of frequency change by introducing a 3-min zeroing period (i.e., no stimulation) in-between frequency changes of alternating EF; frequencies were changed from 0.2 Hz to 200 kHz or vice versa in similar orders as previous experiments (**Figures 3**, **4**). Only a few random neurons showed spiking activities, and no synchronized oscillations were found (data not shown).

### Hypothesis of Coordinated Stimulation by Alternating EF

**FIGURE 8:**
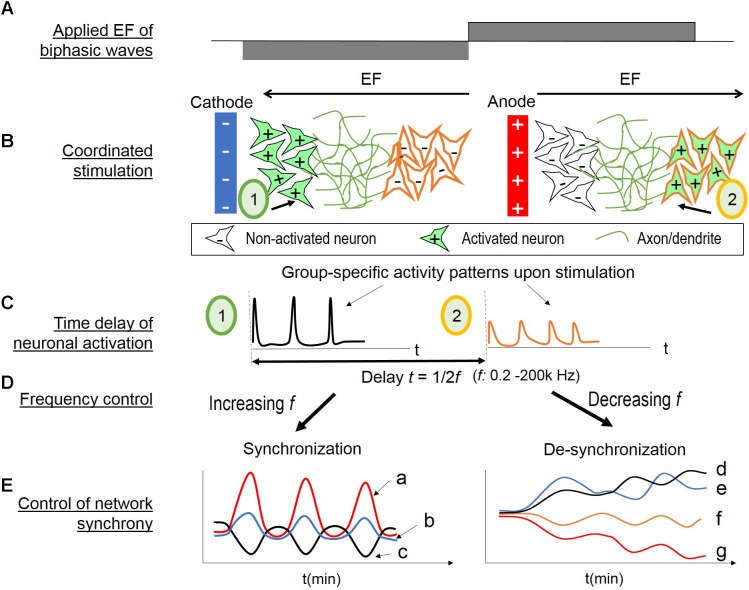
Hypothesis of coordinated stimulation by alternating EF of changing frequencies. **(A)** Biphasic wave stimulation is applied. **(B)** Two populations (1 and 2) with different EF threshold are located at different distances from a nearby electrode. When the electrode is cathode (*left*), population 1 is activated (in *green*, + indicating depolarization) and population 2 non-activated (in *white*, – indicating no change or hyperpolarization). When the electrode turns to anode (*right*), population 1 is inactivated and population 2 is activated. **(C)** Calcium transients occur upon neuronal activation (*left* for population 1; *right* for population 2). There would be a time delay of the population activation, as the inverse of two times of the EF frequency. **(D)** Control of the EF alternating frequency provides a tool to alter the time delay, thus associate or dissociate the two neuronal populations’ activities. **(E)** Network synchrony control by EF alternating frequency. (***Left***) Increasing EF frequency provides repetitive stimulus, and the wide span of frequency range activate different sub-populations. Combined, these conditions lead to network synchrony. The initial evoked response to the applied EF depend on neurons’ spontaneous state at the time of stimulus, therefore, resulting in population-specific oscillations with different amplitude or phase patterns **(a, b, c)**. (***Right***) Conversely, decreasing EF frequency could “unbound” the endogenous activities of different neuronal populations. The initial high frequency (i.e., 200 kHz) stimulation suppresses all activities. As the frequency decreases, the timing between neuronal activation increases. Therefore, the subpopulations are less likely to fire together, resulting in divergent oscillation patterns of different amplitude (**d** versus **e** in the early stages, and **f** versus **g**) or different phase patterns (**d** versus **e**).

Based on these findings, we proposed a hypothesis of network synchrony control by applied EF of alternating polarity (**Figure 8**). Applied EF results in the polarization of the membrane of the nearby cells (Jefferys, 1995; Bikson et al., 2004; Radman et al., 2009). In general, neuronal elements are depolarized near cathode and hyperpolarized near anode. However, the spatial distribution of such polarization under a uniform EF is highly variable, depending on cell biophysics and morphologies (Bikson et al., 2004; Radman et al., 2009; Yi et al., 2017). By extending these concepts to a neuronal network, we hypothesized that different populations are depolarized under a same uniform EF, and that as the field polarity changes, the populations switch to the other activation state (i.e., hyperpolarization versus depolarization). Therefore, biphasic EF would result in coordinated stimulation of neuronal populations.

As illustrated in **Figure 8B**, two populations (1 and 2) with different EF threshold are located at different distances from a nearby electrode; the other electrode would be too far away to impose direct effect. When the electrode is cathode (*left*), population 1 is activated (in *green*, + indicating depolarization) and population 2 non-activated (in *white*, – indicating no change or hyperpolarization). When the electrode turns to anode (*right*), population 1 is in-activated and population 2 activated. **Figure 8C** illustrates the resulting calcium transients upon neuronal activation (*left* for population 1; *right* for population 2). There would be a time delay of the population activation, as the inverse of two times of the EF frequency. Control of the frequency (**Figure 8D**) would provide a means to temporally associate or dissociate the two neuronal sub-populations’ evoked activities.

**Figure 8E** illustrates the hypothesized network synchrony control by EF alternating frequency. *In vitro* studies of random cortical networks have shown that repetitive, time stimulation of loosely associated neurons can induce synchronized bursts of the neurons and their neighbors (Tateno and Jimbo, 1999; Shahaf and Marom, 2001). Increasing EF frequency would be analogous to repetitive stimulus with increasingly shorter timing. In addition, the wide range of frequencies could activate many sub-populations of different responsiveness. It would result in network synchrony (**Figure 8E**, left). The initial response to the applied EF would depend on neurons’ endogenous activities, as shown in **Figure 3D**. Moreover, binary responses to EF (activity increase or decrease) would lead to symmetrical phase pattern, as shown in **Figures 3**, **5**. Therefore, group-specific oscillations with different amplitude or opposite phase patterns would be expected (**Figure 8E**, *left*, a, b, c).

Conversely, decreasing EF frequency could dissociate the endogenous activities of different neuronal sub-populations (**Figure 8E**, *right*). High frequency EF is known to suppress neuronal activities (Wagenaar et al., 2004; Chao et al., 2005; Birdno and Grill, 2008), also shown in our studies with the initial 200 kHz stimulation (**Figure 5C**). As the frequency decreases, the timing between neuronal activation increases, and the sub-populations are less likely to fire together, resulting in divergent oscillatory patterns. Population-specific responsiveness could be the different amplitudes, for example, **Figure 8E-f** versus g, or different phase patterns as d versus e.

## Discussion

We presented results of the behavior of a random cortical network under applied electrical field. Each neuron’s activity was captured by calcium live imaging and matched to its physical location in the network. Calcium signal time series were subjected to cluster analysis for unbiased detection of neuronal communities of similar activity patterns. Spatial and temporal associations of neuronal activities revealed large scale, synchronized oscillations of a random network under alternating EF of changing frequencies. EF without polarity change or frequency change failed to produce synchronized activities among neurons. These findings formed the basis of a hypothesized network control mechanism, involving coordinated stimulation of different sub-populations by alternating field polarity. Change of EF frequency was critical for control of the time delay of group-specific activities, by associating or dissociating different sub-populations via frequency increases or decreases, respectively. These novel EF effects on random neural networks provide important understanding of network synchrony underlying brain functions and neuromodulation applications.

### Neural Network Manipulation and System Setup

A thin silk fibroin-based film with embedded gold wires provided the interface system for *in vitro* cortical cultures. Compared to rigid MEA substrates, the flexible and transparent silk film provides greater ease and superb compatibility with *in vitro* neuronal cultures (Tang-Schomer et al., 2014b) as well as *in vivo* brain implants (Kim et al., 2010; Tang-Schomer et al., 2014c). The wire embedding method simplifies interface fabrication compared to the lithographic process for surface electrodes (Tang-Schomer et al., 2014a), with excellent interface stability requiring no additional adhesives or bonding. Regarding signal transmission, the thin silk film (∼5 μm) poses no significant barrier (>90% conductivity) (Hronik-Tupaj et al., 2013). The gold wire provides double layer capacitive charging (Brummer and Turner, 1977) and modifies the ionic composition near the electrode. By applying charge-neural biphasic field, potential pH buildup at the electrode-solution interface would be eliminated and field propagation increased at high frequencies (Wagenaar et al., 2004; Graves et al., 2011). These features support the use of silk film-based neural-electric interface as a suitable system for investigating EF effects on neural networks.

Sorting activities onto source neurons and grouping them based on common behaviors are not trivial tasks (Morin et al., 2005). Recorded electrical signals have superior temporal resolution allowing for temporal correlation analysis, for example, the delay between stimulus and the first evoked pulse. Temporal correlation of these signaling events forms the basis for inferring functional association of distributed neuronal populations. In comparison, the temporal features of calcium signals are less sharp (Robinson et al., 1993), and slow fluorescence imaging further limits the temporal resolution. In this study, we used confocal 3D imaging to maximize captured neurons that took almost 1 min for each *z*-stack. The slow sampling rate precluded us from examining fast events. Faster imaging in future could allow for detailed temporal analysis. Nevertheless, calcium imaging provides undisputable spatial resolution and allows for the signal trace to be mapped to the source neuron. The individually traceable time series have provided a multi-dimensional picture of the network dynamics for each cell at each time point.

We used community detection in functional networks for the unsupervised identification of neuronal communities that, within a given culture, exhibit homogenous fluorescence-based discharge patterns. Community detection is an established area of network analysis (Newman, 2010) and it has been recently used to unravel structural and dynamical properties of complex neuronal networks such as the epileptogenic brain network in patients with drug-resistant epilepsy (Khambhati et al., 2015), circadian-clock-related networks of neurons in the suprachiasmatic nucleus (Park et al., 2016), and networks of ganglion cells from retina (Billeh et al., 2014). Community detection algorithms, though, are typically applied to large (i.e., more than 1000 nodes) networks while the LA was used in our study on small-size (i.e., up to 70 nodes) neuronal networks. As the size of the network grows, however, the community detection remains feasible. Locally-greedy, resolution-adaptive algorithms (Bassett et al., 2013) and null models (Newman, 2010) are available to guarantee fast neuron clustering, while avoiding the detection of spurious and statistically nonsignificant communities.

### Point and Distributed Electrical Fields for Network Stimulation

Point-source pulse stimulation is the most commonly used modality in neurophysiology studies. Specific stimulation frequencies have been associated with functional responses, for example, hippocampal resting activity (5 Hz), long-term potentiation (LTP, 100 Hz, 1–3 s), long-term depression (LTD, 0.5–5 Hz for 5–30 min), or homeostatic synaptic depression (3 Hz, 12–24 h) (Larson et al., 1986; Staubli et al., 1999; Malenka and Bear, 2004; Goold and Nicoll, 2010). However, it is unclear how the parameters developed with defined CNS pathways can be applied to a random network of *in vitro* cortical culture. In fact, the wide range of frequencies tested in our experiments failed to produce correlated activities of among neurons in culture.

Studies of *in vitro* cortical networks showed that evoked responses depend on the neuron’s endogenous activities (Jimbo et al., 1999; Wagenaar et al., 2005) and that time varying stimulus is more effective in inducing bursting spikes of neuronal ensembles (Shahaf and Marom, 2001; Bakkum et al., 2008b). A series of studies by Jimbo and colleagues reported long-lasting (∼30 min) binary responses of stimuli-induced, large-scale (ensemble) changes in connectivity (Maeda et al., 1995; Jimbo et al., 1999; Tateno and Jimbo, 1999). It was shown that for a given site of tetanic stimulation, all the activated neurons either increase their responsiveness (potentiation) or decrease their responsiveness (depression) to the stimulus (Jimbo et al., 1999). Our results are consistent with these reports as we showed that (i) there were sub-population-specific responses to the same stimulus, (ii) the initial evoked responses were dependent on group-specific endogenous activities prior to the stimulation, and (iii) the evoked response was binary (i.e., activity increase or decrease) upon stimulation. In addition, our study showed that time varying frequency, but not constant frequency, produced synchronized network activities.

However, evoking network synchrony with point stimulation would require pre-selecting a site for stimulation, matching the initiating stimulus with the selected neuron’s responsiveness, and tailoring stimulus time series for each affected neuron (or ensembles) in the network. These tasks would be daunting, if not impossible, for a random network. Alternatively, distributed EF stimulation used in our study would allow different sub-populations to be activated simultaneously. Although speculative at this stage, it is worth noticing that if stimuli-induced changes are operated under the pathway-specific principle as suggested by Jimbo et al. (1999), then group-specific responses would be paced by network-level changes. Hence, intrinsic activity fluctuations would be expected to ride along a slower wave of network oscillation. Indeed, in both scenarios of alternating EF stimulation in our study, the different group-specific calcium signal time series showed oscillatory patterns in synchrony with one other at a time scale (tens of minutes) much longer than previously reported neuronal activities (i.e., milliseconds). The oscillatory patterns were not precisely aligned with the temporal changes of stimulus, in part due to the crude temporal resolution (in minutes) used in the study. Nevertheless, it is interesting to note that the network-level oscillation had a wave length of approximately 15 min, about one round of frequency changes of 6 min per frequency. Focal stimulation studies showed that periodic stimulus can be used to phase lock bursting activities of a local network (Maeda et al., 1995; Darbon et al., 2002). Accordingly, our results imply that the network may not only respond to EF frequency and duration, but also to the change of EF frequency over a longer time scale.

### Network Synchrony Under EF of Alternating Polarity at Changing Frequencies

The most interesting finding of this study is control of network synchrony with EF of alternating polarity and changing frequencies. Field polarity change was found to be essential for network synchronization, as monophasic fields of the same frequency changes failed to produce correlated activities among neurons (**Figure 7**). Other systems have shown that temporal coordination of distributed neuronal activities establishes network synchrony (Singer, 1999). We hypothesized that the alternating field polarity could introduce a time delay of half period of the biphasic wave, and therefore, temporally coordinate the stimulation of different neuronal sub-populations in a network (**Figure 8**). As illustrated in **Figure 8B**, this hypothesis assumes that different sub-populations are activated according to the nearby electrode’s status as cathode or anode. *In vitro* cortical cultures consist of many neuronal types with a wide range of sensitivities to EF as low as 5 mV/mm (Jefferys, 1995; Bikson et al., 2004). In general, neuronal elements are depolarized near cathode and hyperpolarized near anode. However, the spatial distribution of such polarization is modified by a neuron’s complex morphology, summation of which would lead to either somatic depolarization or hyperpolarization (Yi et al., 2017). Therefore, it is reasonable to assume different activation state of sub-populations under a uniform EF, which depends on neuron-specific features. Neuronal sensitivities to different stimulus shapes have been examined in studies by Wagenaar et al. (2004) by using MEAs in cortical cultures. It was found that the transition between the positive and negative phases is the most effective stimulus compared to other pulse shapes. It is possible that different sub-populations have different sensitives to the phase transition rate and are activated at different time points throughout the wide range of frequency change span.

Another key factor is change of polarity alternating rate, or the time differential of the EF frequency. Alternating EF of a constant frequency did not produce correlated activities among neurons, neither did introducing resting-periods in-between EF frequency changes. These results suggested that change of EF frequency was necessary for inducing large scale, synchronized activities of neurons. The hypothesis of coordinated stimulation by EF frequency-dependent time delay could explain these findings (**Figures 8D,E**). Increasing the frequency of the biphasic wave would increase temporal correlation of the activation of different sub-populations. Conversely, as the EF polarity change rate decreases, different sub-populations are less coordinated, resulting in more divergent activities with group-specific oscillatory patterns. Neuronal sensitivity to stimulation frequency are widely reported to affect neural network activities, including adaptation (Eytan et al., 2003), phase-locking (Leondopulos et al., 2012), conduction block (Kilgore and Bhadra, 2004), and rhythm modulation (Birdno and Grill, 2008). This is the first report of network response to continuous frequency change.

It seems unnecessary to increase the frequency up to 200 kHz, as high frequency stimulation is known to pace networks to refractory state (Chao et al., 2005; Wagenaar et al., 2005). When we designed the study, we chose to test the broadest range of frequencies that our stimulator can provide. We had also reasoned that the higher frequencies beyond the membrane polarization threshold would, essentially, act as a direct constant field. Our results showed that the synchronized activity persisted at higher frequencies. Given the abovementioned literature, it is unlikely that these activities are direct result of high frequency stimulation. According to our hypothesis, these behaviors would depend on the *history* of frequency change. Further studies focusing on the regime of 2–200 k will be needed to clarify the input/output correlations and determine the upper limit of EF frequency. It is possible that the induced synchronous oscillation could persist after reaching a frequency threshold. It will also be important to test a wider range of parameters, including frequency range, duration, order of frequency change, etc.

The study had focused on neurons adjacent to the electrode (within 750 μm). Less response would be expected of the neurons in distant areas as there would be little voltage gradient in the middle of the culture. At present, it is unclear whether synchronous oscillation near the electrode can propagate to other part of the network. Detailed mapping of neuronal communities in relation to field polarity and strength will provide insights on how the network communicates changes.

### Implications for Functional Modulation of Neural Networks

Synchronous oscillatory activity in the cerebral cortex plays a crucial role in implementing complex brain functions (e.g., memory, cognition) as well as encoding information (Buzsaìki, 2006). Numerous studies, both *in vitro* and *in vivo*, have focused on the mechanisms that sustain oscillations and their synchronization as well as on the relationship between neural oscillations and network dynamics, e.g., for a review, see (Buzsaki and Draguhn, 2004). Abnormal increments in synchronization are reported as a key component in chronic neurological disorders, e.g., Parkinson’s disease and epilepsy, and in the impairment of decision-making capabilities (Ross et al., 2013; Tan et al., 2013; Broggini et al., 2016; Cao et al., 2016). Our study demonstrates that widespread oscillations can be induced in a neural population *in vitro* by using a coordinated electrical stimulation paradigm with biphasic rectangular waves. Our solution may be used to recreate oscillatory conditions *in vitro* with a fine spatial resolution. The system provides an easy-to-use testbed for reproducing pathological oscillatory activities in large neural populations as well as studying the effects of exogenous inputs (e.g., chemical compounds or novel neuromodulation approaches) on neural oscillations.

Furthermore, noninvasive brain stimulation with electrical or electromagnetic waves provide effective neuromodulation interventions for treating a range of neurological and psychiatric disorders, including deep brain stimulation (DBS) for Parkinson’s, essential tremor, and dystonia (Gross and Lozano, 2000; Ferrucci et al., 2008), transcranial direct or alternating current stimulation (tDCS or tACS) therapies for Alzheimer’s disease (Ferrucci et al., 2008) and stroke (Hummel et al., 2005) and transcranial magnetic stimulation (TMS) for depression. In particular, tACS applies a weak sine-wave electric current to the scalp to identify a cortical oscillation pattern associated with a specific aspect of cognition or brain function and then to apply frequency-matched stimulation with concurrent assessment of changes in the targeted behavior (Frohlich, 2014). The choice of stimulation frequency has relied on the simple assumption that the stimulation frequency applied is the frequency that will induce or enhance in the network, with an implicit assumption of the linearity of the stimulated system. However, there is little reason to assume that the interaction of periodic stimulation with endogenous cortical network dynamics follows the same rules (Frohlich, 2015). Indeed, the cortical network used in the study demonstrated sub-population specific oscillatory patterns that were susceptible to entrainment by applied EF of a wide range of frequencies. These findings suggest that varied EF with controlled polarity and frequency changes could be a more effective means for neuromodulation than a paradigm with fixed polarity and frequencies. For example, applied alternating EF with increasing frequency may induce entrainment of different endogenous oscillators to synchronize activities of different brain areas. Conversely, alternating EF with decreasing frequency starting from refractory high levels may be used to suppress undesirable synchronized activities in some pathological conditions, and “re-tune” the activities back to the level of background oscillations.

However, in order to translate the study’s findings into effective neuromodulation application, specific parameters need to be identified, for example, the upper limit of the EF frequency and time variants of frequency change. In particular, the study only examined square waves and the effects of other waveforms such as sinusoidal inputs are unknown; though other studies have suggested different entrainment properties on cortical oscillations by distinctive waveforms (Hutt et al., 2018). Cell culture-based models provided by this study and other systems (Frohlich, 2015), combined with computational and mathematical simulations (Hutt et al., 2018), will be powerful tools to test different neuromodulation paradigms.

### Limitations of the Study

A major limitation is that different stimulation paradigms were tested with different cell cultures, due to technical constraints of calcium dye use and live imaging, and to avoid potential residual effects of serial stimulations; therefore, direct comparison of different stimulation protocols was lacking. We had made assumption that each cortical cell culture is representative of all mixed cortical cultures, as a common practice with primary cortical culture-based studies. For our analysis, we normalized the fluorescence signals with the global average and standard derivation to prevent culture-specific biases. In addition, the hypothesized mechanism does not rely on neuronal cell composition or network topology that are the major variables between different cultures. Another limitation is the crude temporal resolution due to the 1 min imaging interval (due to slow *z*-stack confocal imaging) that prevented us from making more precise correlations between the stimulation conditions with neuronal responses. Future studies need faster imaging protocols.

## Author Contributions

MDT-S designed the study and performed the experiments in the laboratory of David Kaplan at Tufts University. MDT-S analyzed and interpreted the data at UConn Health and wrote the manuscript at the Jackson Laboratory for Genomic Medicine. TJ assisted with image processing. SS performed the computational analysis and contributed to the preparation of the manuscript.

## Conflict of Interest Statement

The authors declare that the research was conducted in the absence of any commercial or financial relationships that could be construed as a potential conflict of interest.
